# Giant cell tumours in fingers among the Inuit population in Greenland

**DOI:** 10.3402/ijch.v75.31285

**Published:** 2016-04-05

**Authors:** Nick Duelund, Kjeld Hougaard

**Affiliations:** Surgery Department, Queen Ingrids Hospital, Nuuk, Greenland

**Keywords:** orthopaedic hand surgery, Arctic, native, benign tumour, slow growing

## Abstract

**Objective:**

Giant cell tumours (GCTs) of the tendon sheets in fingers are rare. We therefore find it of interest to report on 5 cases identified in the Inuit population in Greenland within 16 months prior to this study.

**Material and methods:**

The Inuit account for 56,000 people of the total population in Greenland. From November 2010 to 16 months prior to this study, we diagnosed 5 cases (0.6% of all orthopaedic operations) with a GCT of the flexor tendon sheet of a finger. The patients were aged between 10 and 54 years, and 4 were women. All of them had noticed slow-growing tumours over 3 or more years and were referred for a suspected ganglion.

**Results:**

In two cases, the tumour was located at the distal interphalangeal (DIP) joint in the thumb and in one case at the third finger. Two other patients had tumours at the metacarpophalangeal (MCP) joint of the third finger and the thumb, respectively; one of these two had a communicating tumour to the DIP joint. The last patient had two tumours on the same finger, one at the MCP joint and the other at the DIP joint. In one case, the tumour had also eroded the cortex of the first phalanx of the thumb, and the largest tumour measured 5 cm.

**Conclusion:**

GCTs of the flexor tendon sheets in fingers are rare. It could be a coincidence that we have seen 5 cases within a short period of time. It is not possible to identify past cases through a register. A tumour in a finger is not the most common location for a ganglion, especially not at the DIP level. Therefore, a large tumour at this location is more likely to be a GCT.

Giant cell tumours (GCT) in the tendon sheets in fingers are benign, slow-growing tumours. They originate from the synovium in the tendon sheets (1).

This type of tumour is the second most common type of tumour found in the hand. It is surpassed only by ganglion cysts (1,2). The aetiology is unknown. There are theories about various aetiologies; among them are reactive, hyperplastic or neoplastic aetiologies of which the first 2 are commonly accepted (3)

The treatment of choice is operative removal, but the recurrence rate is high, up to 44% has been reported (4).

The typical appearance is in the age group between 30 and 40 years, slightly more common among women (5). Except ganglion cysts, tumours in the hand are rare.

A study conducted by Edinburgh University Medical School in 1990–1997 showed 8 cases with GCT each year (on average) in a population of 400,000 (6). We therefore find it of interest to report 5 cases of GCT in fingers in the small Inuit population of Greenland within 16 months prior to this study (the first 4 within 12 months).

## Material and methods

In the period from November 2010 to 16 months prior to this study, we diagnosed 5 patients with GCTs in the tendon sheets of fingers (0.6% of the total number of orthopaedic operations within a year). Patients were aged between 10 and 54 years, and 4 were woman. Two of the patients have had a previous operation more than 3 years ago in their home village in connection with the yearly visit of an orthopaedic surgeon. The operation had been performed under local anaesthesia for a suspected synovial ganglion. Microscopy was performed in one of the two cases. This confirmed it was a GCT. Both had recurrence of the tumour within months.

However, all 5 patients were admitted to the capital city suspected to have synovial ganglions, and all of them had a medical history of a slow-growing tumour over several months or years.

## Results

During the operation, we found the tumour adherent to the flexor tendon sheet in 2 cases; the first patient had a tumour at the metacarpophalangeal (MCP) joint of the right third finger, and the second patient had 2 tumours, one at the MCP joint and the other at the distal interphalangeal (DIP) joint, both in the left third finger. In one case, the tumour was adherent to the extensor tendon but continued around the finger to the flexor tendon sheet at proximal interphalangeal (PIP) joint level of the third finger. In the last 2 cases, the tumour was adherent to the extensor tendon, DIP joint of the left third finger and DIP joint of the right thumb.

**Fig. 1:**
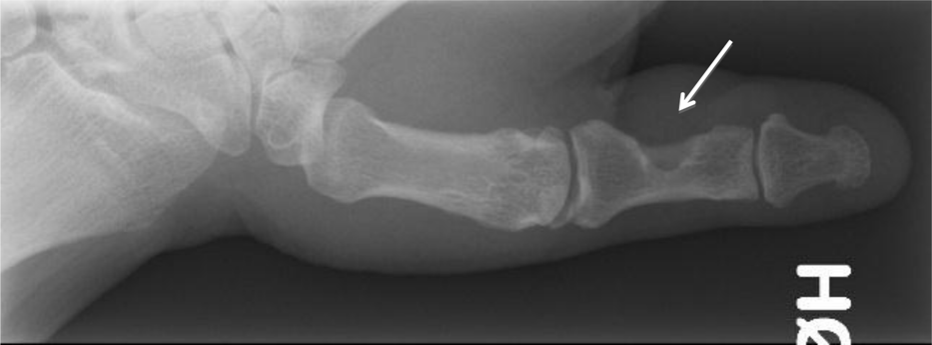
Bony destruction of the first phalanx of the thumb (arrow).

**Fig. 2:**
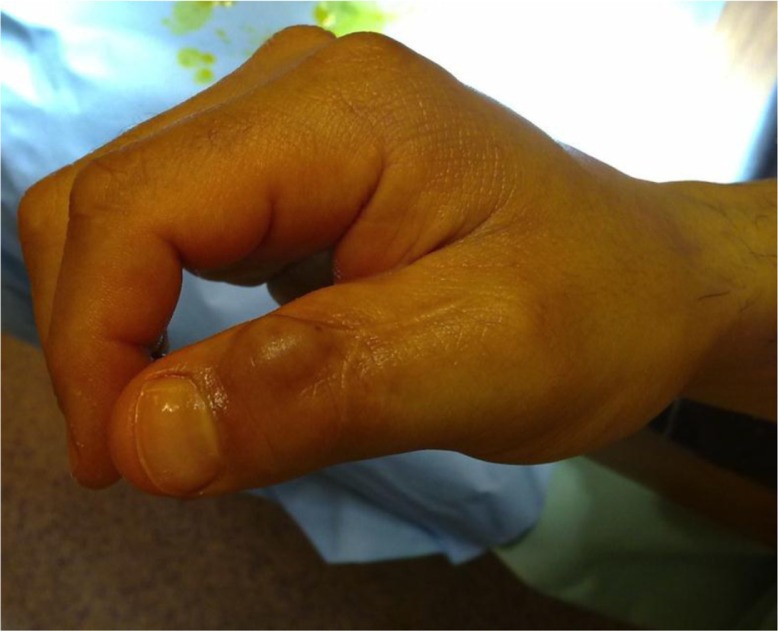
Bulging tumour over the DIP joint. Scars from the first operation.

**Fig. 3:**
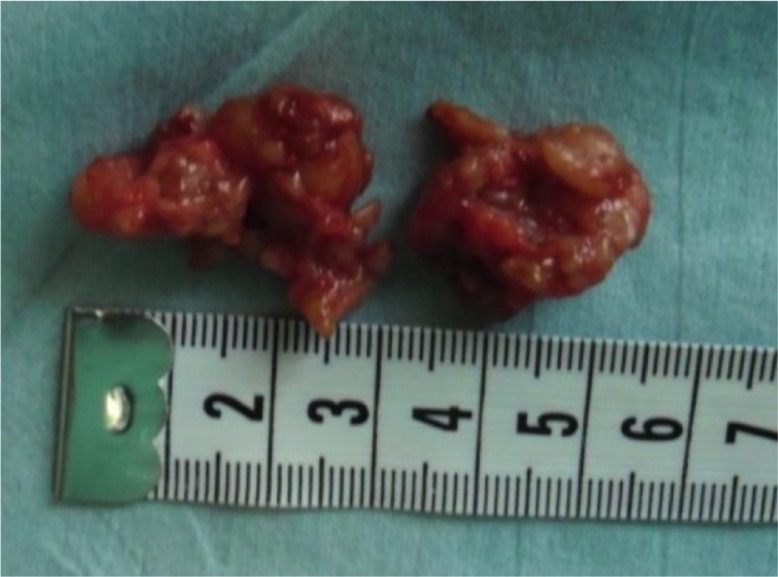
A typical brownish-red appearance of a giant cell tumour.

In one of the patients with recurrence of the tumour, bony destruction had appeared on the first phalanx of the thumb (Fig. 1). The tumour was also bulging under the skin over the dorsal side of the DIP joint where the scar from the previous operation could be seen (Figs. 2 and 3). In all 5 cases, histological examination confirmed it was GCT.

During a follow-up 3 years after surgery, 3 of the 5 patients clinically had recurrence of excised tumours, of which one has had another surgery where it was histologically confirmed to be a GCT.

## Discussion

In the 16 months prior to this study, we have operated 5 Inuit patients in Greenland with a GCT in fingers. The aetiology of tumour development is unknown, so it may be a coincidence. A genetic disposition or exposition from the environment may be possible. All 5 cases were found among those born to Inuit parents in Greenland.

It was impossible to trace other cases of GCT or ganglion cysts treated in the past. Ganglion cysts present themselves mainly at the dorsal side of the wrist (60–70%) (7).

Slow-growing finger tumours, especially appearing at DIP levels in fingers (which is not an uncommon place for GCTs) therefore has to be suspected to be a GCT instead of ganglion cysts, which is not an uncommon place for GCT.
